# A Review of the Ocular Phenotype and Correlation with Genotype in Poretti–Boltshauser Syndrome

**DOI:** 10.3390/medicina61050881

**Published:** 2025-05-12

**Authors:** Won Young Moon, Sanil Shah, Nervine ElMeshad, Samantha R. De Silva

**Affiliations:** 1 Oxford Eye Hospital, Oxford University Hospitals NHS Foundation Trust, Headley Way, Headington, Oxford OX3 9DU, UKnervine.elmeshad@ouh.nhs.uk (N.E.); 2 Nuffield Laboratory of Ophthalmology, Nuffield Department of Clinical Neurosciences, University of Oxford, Oxford OX3 9DU, UK

**Keywords:** Poretti–Boltshauser syndrome, *LAMA1*, laminin, high myopia, strabismus, retinal dystrophy

## Abstract

*Background and Objectives*: Poretti–Boltshauser syndrome (PBS) is a rare, autosomal recessive disorder caused by pathogenic variants in the *LAMA1* gene, resulting in laminin dysfunction. This manifests as a cerebellar malformation with cysts, and patients present with developmental delay and ataxia; however, ocular features are not well-characterised. We aimed to summarise the ocular phenotypes of PBS based on cases reported in the literature. *Materials and Methods*: A literature search was conducted on Medline, Embase, and PubMed on PBS and its ocular associations. Genetically confirmed PBS cases were reviewed, and genotype–phenotype correlations were investigated. *Results*: Comprehensive reporting of genotypes and associated systemic and ocular phenotypes was available in 51 patients with PBS, who had 52 distinct variants in *LAMA1*. Most patients carried homozygous variants. The most common genotype was a c.2935delA homozygous mutation, followed by the c.768+1G>A; c.6701delC compound heterozygous mutation. High myopia was the most common ocular phenotype (*n* = 39), followed by strabismus (*n* = 27) and ocular motor apraxia (*n* = 26). A wide range of other ocular manifestations, including retinal dystrophy, retinal neovascularisation, retinal detachment, strabismus, nystagmus, optic disc and iris hypoplasia, were reported. Patients with the same genotype exhibited variable expressivity. *Conclusions*: PBS has a broad ocular phenotypic spectrum, and characterisation of this variability is important for making an accurate diagnosis and informing genetic counselling.

## 1. Introduction

Poretti–Boltshauser syndrome (PBS) is a rare genetic condition characterised by non-progressive cerebellar dysplasia and is inherited in an autosomal recessive manner [1]. It is caused by mutations in the *LAMA1* gene, which encodes the alpha subunit of laminin, the major component of cell basement membranes [1]. Laminin is also responsible for cell signalling through its C-terminal, which allows cells to migrate and adhere to the basement membrane of tissues during development [1]. Over 40 mutations in *LAMA1* have been reported in the literature, including frameshift, multiexon deletions or duplications, nonsense and splice site variants, and missense variants [1,2]. Most affected patients have biallelic homozygous *LAMA1* mutations, although a few patients carry heterozygous variants [2]. *LAMA1* mutations have been reported in people of different ethnicities, including Europeans, Asians, and mixed ethnicity backgrounds [3], and parental consanguinity was reported in 6% of patients in one case series [2]. The systemic phenotypes of affected patients with the same genotype are often homogeneous, although they may vary even with the same *LAMA1* variant [2].

**Figure 1:**
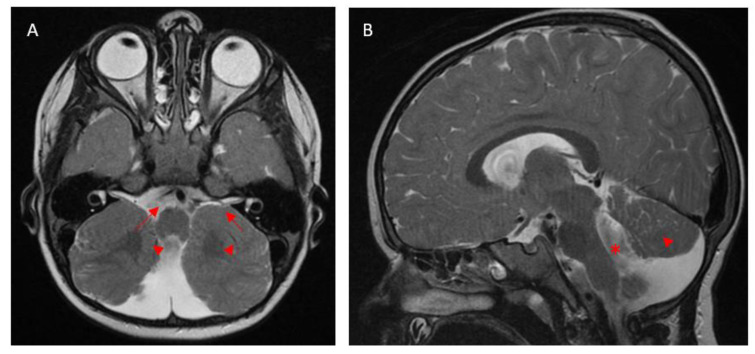
(**A**) Axial and (**B**) sagittal views of a T2-weighted MRI brain scan with gadolinium contrast enhancement. The axial view (**A**) shows hindbrain malformation with severe cerebellar dysgenesis affecting both cerebellar hemispheres. The superior cerebellar peduncles (marked with arrows) are thin and bowed out laterally, and the cerebellar vermis is malformed and hypoplastic (marked with arrowheads). The sagittal view (**B**) shows vermis hypoplasia (marked with arrowhead) with a widened dysmorphic fourth ventricle (marked with *).

Most individuals affected by PBS present before the age of 6 months, usually with developmental delay [2]. Associated clinical features include manifestations of cerebellar dysfunction such as dysmetria, oculomotor apraxia, cerebellar ataxia [2], and truncal ataxia, and other systemic features such as bilateral toe syndactyly have been reported. Most affected children have a delay in cognition, language, and motor development [1] and are noted to have a varying degree of intellectual impairment, the severity of which is not correlated with the degree of brainstem and cerebellar malformation [2]. MRI scans show associated characteristics, such as enlarged and elongated fourth ventricles and hypoplasia of the cerebellar vermis [2] (Figure 1). PBS can be differentiated from other cerebellar dysplasias by the absence of muscular dystrophy and lack of supratentorial abnormalities [2]. The cerebellar dysplasia also tends to affect the entire cerebellum in contrast to other cerebellar dysplasia syndromes [2]. The absence of cerebellar cysts does not exclude PBS [2]. In addition, genetic testing has revealed more cases of PBS that were previously diagnosed as other cerebellar dysplasia syndromes.

Compared to the systemic features of PBS, far less is known about its ocular manifestations, and in some patients, PBS has been reported to present with ocular manifestations without systemic signs, making the diagnosis challenging [1]. We review the current literature to examine the ocular phenotypes of PBS in association with reported genotypes.

## 2. Materials and Methods

**Figure 2:**
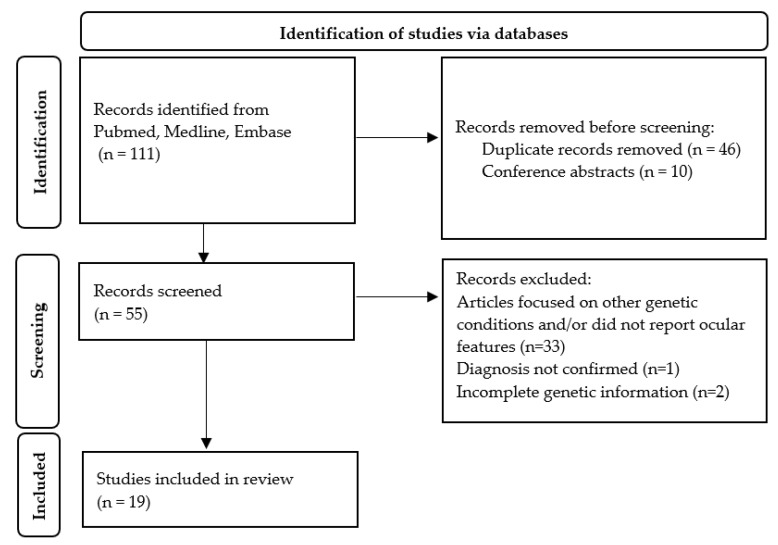
An adapted PRSIMA diagram illustrating the article selection process. This diagram has been adapted from Page MJ, McKenzie JE, Bossuyt PM, Bourton I, Hoffmann TC, Mulrow CD, et al. The PRISMA 2020 statement: an updated guideline for reporting systematic reviews. BMJ 2021;372:n71. Doi: 10.1136/bmj.n71 [4].

Medline, Embase, and PubMed databases were used to conduct a literature search, with the keywords “Poretti–Boltshauser syndrome” and/or “*LAMA1*”. Articles selected were limited to those in the English language published up to and including November 2024, that reported cases of PBS confirmed on genetic testing with details of ocular manifestations. One hundred and eleven articles were identified. Duplicate articles (*n* = 46) and conference abstracts (*n* = 10) were excluded. Fifty-five articles were screened further, of which 36 papers were excluded for the following reasons: articles that focused on other genetic conditions and/or did not report ocular features (*n* = 33), the diagnosis was not confirmed (*n* = 1), or there was incomplete genetic information (*n* = 2). Nineteen articles in total were therefore included for review in this paper (Figure 2). 

## 3. Results

### 3.1. Demographics and Clinical Presentation

Fifty-one patients with a genetically confirmed diagnosis of PBS with ocular involvement have been reported in the literature to date (Table S1). Twenty-seven patients were female and twenty-four were male. Most cases were reported in Western Europe (*n* = 13) followed by Asia (*n* = 8), Eastern Europe (*n* = 6), the Middle East (*n* = 4), Africa (*n* = 3), North and South America (*n* = 3), or were of unknown geographical location (*n* = 10). On average, patients presented at an age of 6 months or younger (*n* = 21). Visual acuity was reported in seven patients (14 eyes). This ranged from LogMAR 0 to 1.1 in both eyes; the mean visual acuity was 0.5, and the median was 0.6. The presence of optic disc atrophy or hypoplasia was noted in six cases (Table 1), and iris hypoplasia was reported in one case.

### 3.2. Myopia and Its Associated Features

**Figure 3:**
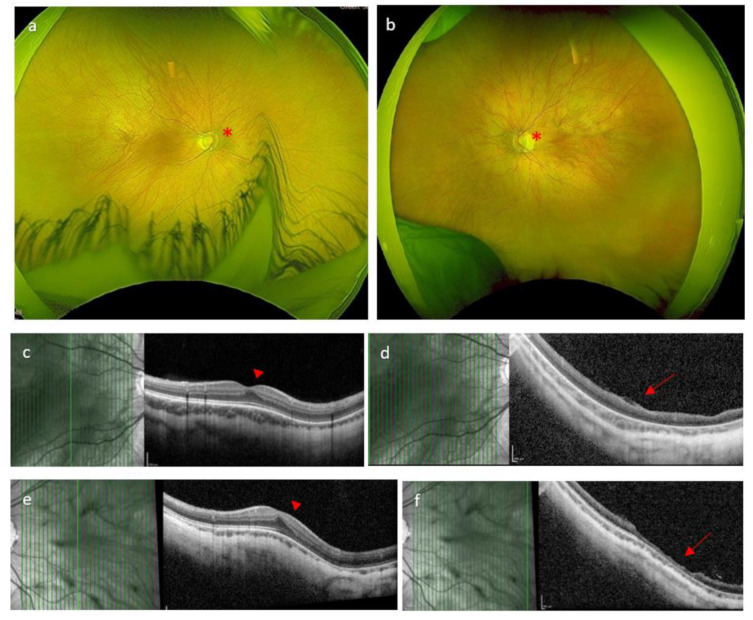
Multimodal retinal images. Multimodal retinal imaging of a 10-year-old girl with high myopia and significant anisometropia with a refraction of −4.25/−2.50 × 180 in the right eye and −13.25/−1.75 × 180 in the left eye. Optos widefield images show peripapillary atrophy (marked with *) and a tigroid fundus pattern in both eyes (**a**,**b**). In the right eye OCT, the retinal layers at the fovea are preserved (marked with arrowhead) (**c**), and there is disruption of the inner layers of the temporal part of the macula (marked with arrow) (**d**). In the left eye OCT, there is a mildly reduced foveal depression (marked with arrowhead), dome-shaped macula (**e**), and loss of inner layers of retina in the temporal macula (marked with arrow) (**f**).

Thirty-nine patients had myopia or high myopia ranging from −3 to −20 dioptres. The presence and the severity of myopia did not correlate with specific variants. High myopia related ocular manifestations included long axial length [5], macular heterotopia [5], optically empty vitreous veils [3], diffuse peripheral thinning extending to the temporal part of the macula [3], foveal hypoplasia [3], atrophic retinal holes [6], lattice degeneration [6,7], tigroid fundus and Foster Fuchs spots [8], and macular holes [9,10] (Figure 3). Two patients had unilateral cataract [11,12] and three cases had bilateral cataracts [5,13,14].

### 3.3. Strabismus and Ocular Motility Dysfunction

Strabismus was one of the most common ocular manifestations in PBS patients. Twenty-seven patients were reported to have strabismus, and nystagmus was reported in seventeen patients. Strabismus and nystagmus due to poor vision were the only ocular manifestations in some cases, and these patients had differing variants [2,15,16]. Two patients had amblyopia. Esotropia was the most commonly reported strabismus. Others were limited supraduction [7], cross fixation [13], and intermittent bilateral exotropia associated with monocular fixation preference [16]. Other ocular motility dysfunctions, including saccadic intrusions [7] and impaired pursuit [9], were reported. Ocular motor apraxia was reported in twenty-six patients.

### 3.4. Retinal Dystrophy

A retinal dystrophy was diagnosed in sixteen patients and supported by electrodiagnostic testing (EDT) in eleven patients, although the details of the results were not specified in two patients [7]. The results of EDTs revealed a predominance of cone involvement, with cone dystrophy and cone–rod dystrophy of varying severity. Two individuals were diagnosed with mild cone dystrophy, with one exhibiting atrophy just temporal to the macula [1] and the other reported to have a normal fundus appearance [5]. Six patients had cone–rod dystrophy, with four of these showing chorioretinal atrophy in the macula as well as peripheral retina, predominantly in the temporal retina [1]. Short-wavelength responses were preserved in three patients with cone–rod dystrophy who underwent S-cone electroretinogram (ERG) testing [1]. Additionally, in three patients with more severe cone–rod dysfunction, there was bone spicule pigmentation as well as macular and temporal retinal atrophy in keeping with a more extensive retinopathy [17]. The distribution of pigment changes varied, with some cases exhibiting central involvement while others had peripheral pigmentation [17].

ERG testing also supported the presence of cone bipolar cell dysfunction. In three patients with cone–rod dystrophy, ERG recordings showed a significantly reduced b/a wave ratio and slightly diminished a-wave response, indicating cone bipolar cell dysfunction alongside cone photoreceptor dysfunction [1]. In contrast, under dark-adapted conditions, these patients displayed relatively normal b/a ratios with reduced a-waves, suggesting rod involvement but intact rod bipolar cell function [1].

Familial exudative vitreoretinopathy (FEVR), like peripheral retinal avascularity, was observed in three cases, two of which developed retinal neovascularisation [1,6], and one of the latter also had a progressive cone–rod dystrophy [1]. More recently, PBS was diagnosed in a 24-year-old patient with a coats-like exudative vitreoretinopathy who had a poor response to treatment [14]. Retinal avascularization and subsequent disruption of inner retinal layers in PBS have also been linked to reduced b-wave amplitudes, particularly under photopic conditions, supporting cone photoreceptor and bipolar cell involvement [5].

### 3.5. Genotype–Phenotype Correlations in LAMA1

Twenty-two patients reported in the literature had homozygous mutations in *LAMA1,* and twenty-six had compound heterozygous mutations (Table S1). Most mutations were truncating or missense. The most common *LAMA1* variant reported was c.2935delA, resulting in p.(R979Gfs*45). The most common genotype was biallelic c.2935delA (*n* = 6), followed by c.768+1G>A; c.6701delC (*n* = 4) (Table 2). Of note, 6 out of 15 families from the Mediterranean region carried the c.2935delA variant, suggesting a founder effect in this group [2]. The six patients with c.2935delA variants were reported in Albania, Bosnia, Kosovo, and Spain [2]. They had varying ocular manifestations, with most exhibiting ocular motor apraxia (*n* = 5) and high myopia (*n* = 3) [2]. Retinal dystrophy was observed in two of these cases, although electrodiagnostic tests were not performed to confirm these findings [2]. In patients with the c. 768+1G>A; c. 6701delC variants [7], high myopia, retinal atrophy, and strabismus were consistently observed. Other mutations, including c. 1492delC (*n* = 3) and c. 2816_2817delAT; c. 555T>G variants (*n* = 2) were associated with similar ocular phenotypes, such as high myopia and cone–rod dystrophy [1,5]. The patients with c. 664C>T; c. 2331C>G variants had more severe pathological myopia-related changes, such as FEVR-like peripheral retinal avascularity, as well as chorioretinal atrophy [6].

## 4. Discussion

PBS is a rare genetic disorder associated with *LAMA1* variants, leading to a broad spectrum of systemic and ocular manifestations. This review aimed to characterise the ocular phenotype of PBS and explore potential genotype–phenotype correlations based on data from fifty-one published cases with ocular phenotypes.

### 4.1. Ocular Manifestations and Their Variability

Myopia was the most prevalent ocular finding, affecting thirty-nine of fifty-one patients, with refractive errors ranging from −3D to −20D. Interestingly, the severity of myopia did not correlate with specific *LAMA1* variants, highlighting the possibility of additional modifying influences. Some patients with high myopia also exhibited secondary retinal changes, such as retinal degeneration and macular holes, while others showed relatively preserved retinal structure despite extreme myopia [6,7,18]. These findings emphasise the complexity of PBS-related ocular abnormalities and the potential for progressive retinal complications.

Strabismus (*n* = 27) and ocular motor apraxia (*n* = 26) were also common, further supporting the role of cerebellar dysfunction in PBS. Notably, in some cases, strabismus was the first or only ocular manifestation [13], underscoring the importance of fundus examination of children in all cases. The absence of other overt ocular abnormalities in some patients with strabismus suggests that ocular motor dysfunction may occur in isolation in certain individuals. This finding aligns with the known role of the cerebellum in coordinating eye movements and highlights the diverse presentation of PBS.

### 4.2. Retinal Dystrophy and Electrodiagnostic Findings

Retinal dystrophy was identified in sixteen patients, with EDT performed in eleven cases, revealing predominantly a cone–rod dystrophy. Clinical features included macular and peripheral retinal atrophy, likely resulting from laminin deficiency, which plays a critical role in retinal structure and function [3,19]. However, as with other ocular phenotypes, there was no clear association between specific *LAMA1* variants and the presence or severity of retinal dystrophy.

ERG findings in PBS patients provide additional insights into retinal dysfunction. Whilst most ocular manifestations varied significantly among patients with identical genotypes, electrodiagnostic findings were relatively consistent, suggesting that electrophysiology may serve as a more reliable marker for ocular phenotypic characterization. For example, all cases with the c.1492delC variant demonstrated preserved short-wavelength sensitivity on S cone ERG tests [1]. However, due to limited data, it is unclear whether this finding is specific to this mutation or a broader characteristic of PBS. However, of all the PBS patients reported, only eleven underwent EDT (with a further five patients diagnosed on the basis of fundus appearance). Therefore, early retinal dystrophies may currently be undetected and more prevalent than reported in the literature.

### 4.3. Genotypic Influence and the Complexity of PBS

The most frequently reported *LAMA1* mutation, c.2935delA, was found predominantly in Mediterranean families, raising the possibility of a regional founder effect [2]. However, even among patients carrying this mutation, ocular phenotypes varied widely, ranging from isolated myopia to retinal dystrophy and strabismus [2]. In addition, variable expressivity was observed as one of the two patients with the same truncating variants had a normal fundus, whereas the other had chorioretinal atrophy involving the macula [5]. This variability underscores the challenges of predicting clinical outcomes based solely on genetic findings.

In this review, we found no clear or consistent correlation between genotype and ocular phenotype in PBS. This variability was observed even among patients carrying identical *LAMA1* mutations, suggesting the involvement of additional genetic, epigenetic, or environmental factors in modulating disease expression. *LAMA1* plays a crucial role in several developmental pathways, and laminin, its protein product, is integral to the basement membrane and extracellular matrix of multiple ocular structures, including the retina [19]. Laminin is part of the glycoprotein complex found in Bruch’s membrane as well as several layers of inner and outer retinal layers, including the interphotoreceptor matrix, inner and outer plexiform layer, and internal and external limiting membrane [3,19]. Disruption of laminin can lead to a wide spectrum of ocular abnormalities, as evidenced by the findings in this review.

PBS-related vascular abnormalities further illustrate this complexity. Laminin is essential for retinal angiogenesis, and its disruption can lead to retinal avascularity, astrocytic migration into the vitreous, and subsequent vitreoretinopathy [14]. These changes can manifest as FEVR-like vitreoretinopathy with a persistent hyaloid artery [1] or coats-like vitreoretinopathy [14]. Unlike classical FEVR, which typically features temporally dragged retinal vessels, PBS-associated vascular abnormalities tend to present with vertically or nasally elongated vessels [5]. Given these atypical presentations, genetic testing should be considered in patients with unexplained vitreoretinopathy. Unlike the non-progressive systemic features of PBS, retinal avascularization can worsen over time, potentially leading to neovascularization and retinal detachment, underscoring the need for regular ophthalmologic monitoring [5].

### 4.4. Clinical Mimics of PBS: Joubert and Knobloch Syndrome

Recognising conditions that mimic PBS is essential for accurate diagnosis and management, and Joubert and Knobloch syndromes are among the main differential diagnoses. Joubert syndrome is a genetic disorder that is also characterised by cerebellar malformation [20]. It shares several clinical features with PBS, including developmental delay, hypotonia, intellectual impairment, and ocular manifestations such as strabismus, nystagmus, ocular motor ataxia, and retinal dystrophy [20]. However, Joubert syndrome involves multisystemic involvement, notably including the liver and kidneys, and typically follows a progressive course, whereas PBS is generally non-progressive [20]. In terms of ophthalmic findings, high myopia and retinal atrophy are more commonly associated with PBS, whereas ptosis and ocular coloboma are distinctive features of Joubert syndrome and have not been reported in PBS [20]. Neuroimaging also plays a key role in distinguishing these disorders. PBS is characterised by cerebellar cysts and thinning of the superior cerebellar peduncles [12]. In contrast, Joubert syndrome demonstrates thickened superior cerebellar peduncles and a deepened interpeduncular fossa, together forming the characteristic “molar tooth sign” on axial imaging [5,12,21].

Knobloch syndrome (KNO) is an autosomal recessive disorder caused by mutations in the *COL18A1* gene, which plays a critical role in ocular embryogenesis [17]. Patients with KNO often present with high myopia and its associated complications, including cataracts, retinal detachment, and lens dislocation—the latter has not been reported in PBS [17]. A distinguishing feature of KNO is the presence of occipital skull defects, whereas PBS primarily involves cerebellar anomalies [17]. Additionally, occipital alopecia areata has been described in KNO, whereas alopecia in PBS, when present, typically affects the parietal region [17]. The underlying pathophysiology of alopecia in PBS remains unclear.

### 4.5. Limitations

This review has several limitations. We included reports published only in the English language, of PBS with ocular manifestations. This may limit our understanding of PBS ocular phenotypes in other populations and affect the overall proportion reported of certain ocular features in PBS patients. Due to the rare nature of the condition, we also included all studies that met the inclusion criteria and did not undertake a formal risk of bias assessment, which could have resulted in publication bias. Also, given the number of cases associated with each genotype was small, we were unable to undertake statistical analyses to establish any statistically significant correlations. Furthermore, the number of patients who underwent ERG was also small, and it is therefore difficult to draw firm conclusions from these tests due to inconsistencies in ERG protocols and techniques used across different studies.

### 4.6. Clinical and Research Implications

**Figure 4:**
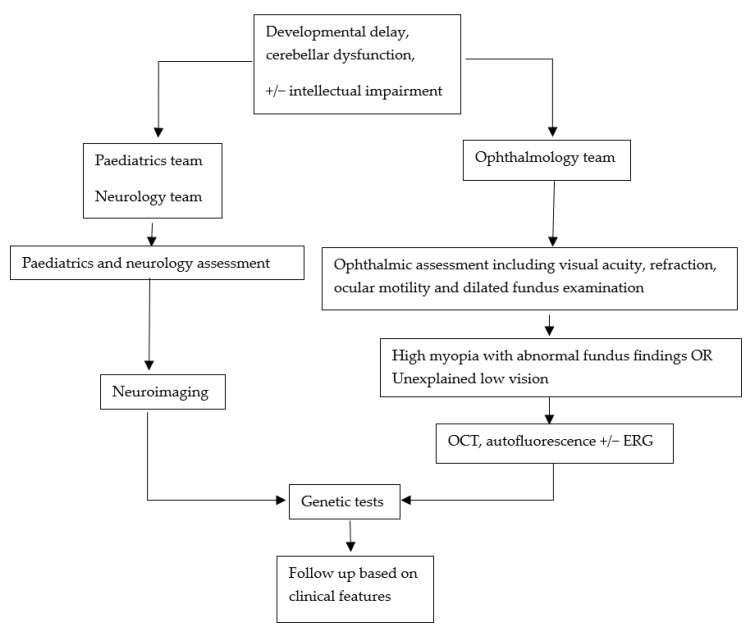
A summary flowchart for the recommended management of children with suspected PBS, highlighting the need for a multidisciplinary team approach and relevant ophthalmic assessments.

This review highlights the extensive spectrum of ocular manifestations in PBS. From a clinical standpoint, the high prevalence of strabismus and ocular motor apraxia suggests that these signs may serve as early indicators of PBS, particularly in infants presenting with developmental delay and cerebellar dysfunction. We recommend that a comprehensive ophthalmic assessment, including cycloplegic refraction and dilated fundus examination, should be routinely undertaken in patients suspected or confirmed to have PBS, the latter to detect early retinal changes. For patients with unexplained low vision and/or abnormal fundus findings, optical coherence tomography and, if possible, electrodiagnostic testing should be considered (Figure 4). Future research should prioritise longitudinal studies to track the progression of ocular features in PBS patients, as many published cases involve young children whose ocular phenotypes may still be evolving. Therefore, the nature of the progression of ocular findings is currently unknown. Additionally, investigating genetic modifiers or the influence of environmental factors could help clarify the variability in ocular presentations and may eventually lead to more tailored management strategies.

## 5. Conclusions

PBS is a rare genetic condition with a wide spectrum of ocular phenotypes. Understanding the ocular phenotype in relation to genotype may assist with genetic counselling, prognostic evaluation, and management of individual cases. For children with developmental delay, reduced vision, or abnormal ocular motility, ophthalmic assessment, including dilated fundus examination, is important. For such children with high myopia and abnormal retinal findings such as chorioretinal atrophy, further assessment with OCT and, if possible, ERG is recommended, and genetic testing should be considered (Figure 4). Advances in genotyping have helped identify cases of PBS in patients who were previously diagnosed as having other conditions. This is also relevant to adults presenting with ocular symptoms, although presentation of PBS in adulthood is rare. The multidisciplinary team approach, with input from paediatrics, genetics, paediatric ophthalmology, optometrists, orthoptists, and eye clinic liaison officer support, is paramount for optimal management of these patients.

## Figures and Tables

**Table 1:** Ocular features associated with PBS in included studies. This table presents the frequencies of reported ocular features observed in 51 patients with PBS across the included studies.

Ocular Manifestations	Number of Patients
Myopia	39
Strabismus	27
Ocular motor apraxia	26
Retinal/chorioretinal atrophy	18
Nystagmus	17
Retinal dystrophy	16
Optic disc hypoplasia or atrophy	6
Cataract	6
Amblyopia	2
Iris hypoplasia	1

**Table 2:** The most frequent variants reported in patients with PBS and ocular features, and their associated ocular phenotypes. Percentages represent the proportion of affected individuals among patients with the same genotype.

Number of Patients with Each Genotype	Variant 1	Variant 2	Myopia	Retinal Atrophy	Retinal Dystrophy	Ocular Motor Apraxia	Strabismus	Nystagmus
*n* = 3	c.1492delC	c.1492delC	100%	100%	100%	66.7%	33.3%	33.3%
*n* = 6	c.2935delA	c.2935delA	50%	0	33.3%	83.3%	33.3%	0
*n* = 4	c.6701delC	c.768+1G>A, c.8557-1G>C	100%	100%	25%	50%	100%	50%
*n* = 2	c.2816_2817delAT	c.555T>G	100%	50%	100%	50%	50%	50%
*n* = 2	c.664C>T	c.2331C>G	100%	0	0	0	100%	100%
*n* = 2	c.3881G>A	deletion of exons 31–32	100%	0	0	50%	100%	0

## Data Availability

All data mentioned in this article are available in the cited articles in the references.

## Supplementary Materials

The following supporting information can be downloaded at: https://www.mdpi.com/article/10.3390/medicina61050881/s1, Table S1: Demographics, genotype and ocular phenotypes of PBS cases with ocular features reported in the literature.

## Abbreviations

The following abbreviations are used in this manuscript:

PBS	Poretti–Boltshauser syndrome
EDT	Electrodiagnostic test
ERG	Electroretinogram
FEVR	Familial exudative vitreoretinopathy
KNO	Knobloch syndrome
