# Supplementary material for: A Review of the Ocular Phenotype and Correlation with Genotype in Poretti–Boltshauser Syndrome
Source: Medicina (Kaunas). 2025 May 12;61(5):881. doi: 10.3390/medicina61050881 (PMC12113114; doi:10.3390/medicina61050881)
Supplement: Supplementary file 1 [file medicina-61-00881-s001.zip › medicina-3611700-supplementary.pdf]

Supplementary Table S1. Demographics and genotype-ocular phenotypes of cases reported in the literature

Patient	Age (yrs)	Gender	Ethnicity/ Country of origin	Variant 1	Variant 2	Visual acuity (logMAR)	Myopia (OD, OS)	Chorio-retinal atrophy	Retinal dystrophy	Optic disc atrophy/ hypoplasia	Cataract	OMA	Strabismus	Nystagmus	Amblyopia	ERG	Ref
P1	42	F	Indian	c.1492delC	c.1492delC	0.6 both eyes	++ (OD −12/+0.50x180 OS −13.50)	+	+	-	-	-	-	+	-	cone-rod dystrophy with preservation of short wavelength	1
P2	40	M	Indian	c.1492delC	c.1492delC	R 0.3 L 0	-15	+	+	-	-	+	+	-	-	cone-rod dystrophy with preservation of short wavelength	1
P3	37	M	Indian	c.1492delC	c.1492delC	0.2 both eyes	++ (OD −13.37/ +0.62x93 OS −4.75/+1.75x60)	+	+	-	-	+	-	-	-	mild cone dystrophy with preservation of short wavelength	1
P4	11	F	Unknown	c.2275_3363del	c.3065delG	R 0.6 L 0.7	++ (OD-11.5 OS -12.5)	+	+	+	-	+	+	-	-	progressive cone rod dystrophy	1
P5	0.3	M	Switzerland	c.8556+1G>A	c.8556+1G>A	N/A	-9	-	-	-	-	+	+	-	-	N/A	2
P6	0.4	F	Albania	c.2935delA	c.2935delA	N/A	-	-	-	-	-	+	-	-	-	N/A	2
P7	0.25	M	Germany	c.4676delA	c.7180C>T	N/A	-13	-	-	-	-	+	+	+	-	N/A	2
P8	0.25--0.5	F	Bosnia	c.2935delA	c.2935delA	N/A	-	-	-	-	-	+	-	-	-	N/A	2
P9	0.25-0.5	M	Bosnia	c.2935delA	c.2935delA	N/A	-	-	-	-	-	-	+	-	-	N/A	2
P10	0.17	M	Germany	c.1774_1775insTTCATAAT	c.6348dupT	N/A	-14	-	+	-	-	+	-	+	-	N/A	2
P11	0.3	F	Kosovo	c.2935delA	c.2935delA	N/A	-13	-	+	-	-	+	-	-	-	N/A	2
P12	0.25-0.3	F	Russia	c.470C>G	g.6999443_6999910del	N/A	-17	-	-	-	-	+	-	-	-	N/A	2
P13	0.25-0.3	M	Spain	c.2935delA	c.2935delA	N/A	-11	-	+	-	-	+	-	-	-	N/A	2
P14	0.25-0.3	F	Italy	c.4663+1G>C	c.1404_1405delAG	N/A	-	-	-	-	-	+	+	+	-	N/A	2
P15	0.25-0.3	M	Turkey	c.2935delA	c.2616delG	N/A	-	-	-	-	-	-	+	-	-	N/A	2

P16	0.17-0.25	F	Italy-England	c.8761C>T	g.6942238_6943401del	N/A	-	-	-	-	-	+	-	+	-	N/A	2
P17	0.17-0.25	F	Italy	c.164A>T	c.2108C>T	N/A	++	-	+	-	-	+	-	+	-	N/A	2
P18	0.3-0.4	M	Turkey	c.3919C>T	c.3919C>T	N/A	-6	-	-	-	-	+	+	-	-	N/A	2
P19	0.3-0.4	M	Albania	c.2935delA	c.2935delA	N/A	-6	-	-	-	-	+	+	-	-	N/A	2
P20	3	F	Iranian	c.588+2T>G	c.588+2T>G	N/A	-13.5	+	-	-	-	-	+	-	+	N/A	3
P21	3	F	Mixed European	c.6345+3G>C	deletion of exons 4-11	N/A	-17	+	-	-	-	-	+	+	-	N/A	3
P22	2.1	M	Mixed European	c.7965-15_7965-3del	c.2988_2989delA	N/A	+	+	-	-	-	-	+	+	-	N/A	3
P23	29	F	Mixed European	c.6701delC	c.768+1G>A, c.8557-1G>C	N/A	-16.5	+	-	-	-	+	+	-	-	N/A	3
P24	23	M	Mixed European	c.6701delC	c.8557-1G>C, c.768+1G>A	N/A	-13.5	+	-	-	bilateral	-	+	-	+	N/A	3
P25	5.5	F	Asian and African American	c.2816_2817delAT	c.555T>G	N/A	-3	-	+	-	-	+	−	-	-	cone rod dystrophy	3
P26	4.5	F	Asian and African American	c.2816_2817delAT	c.555T>G	N/A	-11.5	+	+	-	-	-	+	+	-	cone rod dystrophy	3
P27	2.5	F	Unknown	c.7160 G > T	c.7160 G > T	N/A	-15	+	+	-	bilateral	-	-	-	-	markedly reduced rod and single flash cone responses	4
P28	<0.4	F	Unknown	c.664C>T	c.2331C>G	R 1.0 L 1.1	-16	-	-	-	-	-	+	+	-	N/A	5
P29	<0.4	M	Unknown	c.664C>T	c.2331C>G	R 0.7 L 0.9	-15.5	-	-	-	-	-	+	+	-	N/A	5
P30	21	M	Unknown	c.6701delC	c.768+1G>A, c.8557–1G>C	N/A	++(OD -20 OS -30)	+	+	-	-	+	+	+	-	Retinal dystrophy	6
P31	26	F	Unknown	c.6701delC	c.768+1G>A, c.8557–1G>C	N/A	-19	+	-	-	-	-	+	+	-	N/A	6
P32	8.5	F	Caucasian and native American	c.2160T>A	c.5985_5991del	N/A	-	-	-	-	-	+	-	-	-	N/A	6
P33	0.67	M	? India	c.8184delT	c.8184delT	N/A	++	-	-	-	-	-	-	-	-	N/A	7
P34	7	M	Turkish	c.8192C>A	c.8192C>A	N/A	-	-	-	+	-	-	-	-	-	N/A	8
P35	1.25	F	Unknown	c.767G>A	c.767G>A	N/A	++	-	+	-	unilateral	+	+	-	-	N/A	9
P36	65	F	Japanese	c.1711_1712del	c.1711_1712del	N/A	-	-	+	-	unilateral	-	-	-	-	N/A	10
P37	1	F	? Belgium	c.8446C>T	c.8446C>T	N/A	++ (OD -11 OS -10)	+	-	+	bilateral	-	+	+	-	Normal	11

P38	24	F		c.206G>T	exon 1-3 deletion	R 1.2 L 0.2	++ (OD −10.00 + 0.50 × 127 OS −10.75 + 0.75 × 92)	+	+	-	bilateral	-	-	-	-	N/A	12
P39	18	M	Unknown	c.756delT	c.756delT	N/A	-	-	-	-	-	+	-	-	-	N/A	13
P40	2.5	F	Caucasian	c.8761C>T	g.6942238_6943401del	N/A	-	-	-	-	-	+	+	+	-	N/A	14
P41	2	M	China	c.4579C > T	c.1487dup	R 0.2 L0.1	++ (OD –11.50 OS –13.25)	+	+	+	-	-	+	+	-	Moderately reduced cone and rod responses	15
P42	0.5	M	China	c.6151C > T	c.1494_1504del	N/A	++ (OD –12.25 OS –10.50)	+	+	+	-	-	+	+	-	Severely reduced cone and rod responses	15
P43	0.4	F	China	c.4171_4172del	c.4171_4172del	N/A	++ (OD –15.00 OS –14.00)	+	+	+	-	-	+	-	-	Moderately reduced cone and rod responses	15
P44	Unknown	Unknown	Unknown	c.3597del	c.3597del	N/A	-	-	-	-	-	-	-	-	-	N/A	17
P45	2.5	M	Unknown	c.4702_4703del	c.4702_4703del	N/A	++	-	-	-	-	-	-	-	-	N/A	18
P46	0.17	M	Unknown	c.3881G>A	deletion exons 31-32	N/A	++ (OD -13.5 OS -12)	-	-	-	-	+	+	-	-	N/A	19
P47	0.25	M	Unknown	c.3881G>A	deletion exons 31-32	N/A	++ (OD -12 OS -11.5)	-	-	-	-	-	+	-	-	N/A	19
P48	3	F	White British	c.494delT	c.3053delC	N/A	+	-	-	-	-	+	-	-	-	N/A	20
P49	6.9	F	Carribean/ Gambian	c.281A>G	c.3397C>T	N/A	+	-	-	-	-	-	-	-	-	N/A	20
P50	8	F	White British	c.2344C>T	c.2962delT	N/A	+	-	-	-	-	-	-	-	-	N/A	20
P51	12	M	White British	c.1281C>A	c.2344C>T	N/A	+	-	-	-	-	+	-	-	-	N/A	20

+: presence of phenotype, high myopia: ++, absence of phenotype: -. F: female M: male, OD: right eye, OS: left eye, N/A: not available, If there is only one refraction data point available for each case, this means that the literature reported only one reading from an unspecified eye. OMA: ocular motor apraxia, Ref: references
